# A selective method for optimizing ensemble docking-based experiments on an InhA Fully-Flexible receptor model

**DOI:** 10.1186/s12859-018-2222-2

**Published:** 2018-06-22

**Authors:** Renata De Paris, Christian Vahl Quevedo, Duncan D. Ruiz, Furia Gargano, Osmar Norberto de Souza

**Affiliations:** 1 Business Intelligence and Machine Learning Research Group—GPIN, School of Technology, PUCRS, Av. Ipiranga, 6681, Building 32, Room 628, Porto Alegre, RS, Brazil; 2 Bioinformatics and Biossystems Modeling and Simulation Lab—LABIO, School of Technology, PUCRS, Av. Ipiranga, 6681, Building 32, Room 602, Porto Alegre, RS, Brazil

**Keywords:** Scientific workflow, Cloud computing, Molecular docking, Fully-Flexible receptor model

## Abstract

**Background:**

In the rational drug design process, an ensemble of conformations obtained from a molecular dynamics simulation plays a crucial role in docking experiments. Some studies have found that Fully-Flexible Receptor (FFR) models predict realistic binding energy accurately and improve scoring to enhance selectiveness. At the same time, methods have been proposed to reduce the high computational costs involved in considering the explicit flexibility of proteins in receptor-ligand docking. This study introduces a novel method to optimize ensemble docking-based experiments by reducing the size of an InhA FFR model at docking runtime and scaling docking workflow invocations on cloud virtual machines.

**Results:**

First, in order to find the most affordable cost-benefit pool of virtual machines, we evaluated the performance of the docking workflow invocations in different configurations of Azure instances. Second, we validated the gains obtained by the proposed method based on the quality of the Reduced Fully-Flexible Receptor (RFFR) models produced using AutoDock4.2. The analyses show that the proposed method reduced the model size by approximately 50% while covering at least 86% of the best docking results from the 74 ligands tested. Third, we tested our novel method using AutoDock Vina, a different docking software, and showed the positive accuracy achieved in the resulting RFFR models. Finally, our results demonstrated that the method proposed optimized ensemble docking experiments and is applicable to different docking software. In addition, it detected new binding modes, which would be unreachable if employing only the rigid structure used to generate the InhA FFR model.

**Conclusions:**

Our results showed that the selective method is a valuable strategy for optimizing ensemble docking-based experiments using different docking software. The RFFR models produced by discarding non-promising snapshots from the original model are accurately shaped for a larger number of ligands, and the elapsed time spent in the ensemble docking experiments are considerably reduced.

**Electronic supplementary material:**

The online version of this article (10.1186/s12859-018-2222-2) contains supplementary material, which is available to authorized users.

## Background

According to Eder et al. [1] the average cost of bringing a new drug to market is doubling approximately every 9 years, while a negative impact has been noted in the number of drug approvals by the US Food and Drug Administration. The development of new drugs is a very lengthy and time-consuming process. It also requires substantial investments in technology resources, such as the computational power to store, manage, execute, and analyze simulations on protein-ligand interactions [2, 3]. Thus, new computational methods are needed to aid time reduction and to accurately investigate chemical and biological behaviors of ligands and receptors during the Rational Drug Design (RDD) process [4, 5].

Molecular Docking, which constitutes the second step of the RDD, is an attractive technique to identify and optimize drug candidates because of its ability to quickly screen large libraries of potential leads for identifying native-like poses and filtering out compounds that are likely nonbinders [6, 7]. It has been widely used in pharmaceutical design since structure-based virtual screening has shown to be more economic than experimental screening [7]. To predict the best orientation of a small molecule (ligand), a molecular docking simulation generates several possible poses that a ligand may fit within the macromolecular target (receptor) binding site using a docking software, such as AutoDock4.2 and AutoDock Vina [8, 9]. Each docking software has a search algorithm that generates a set of different binding modes of a protein-ligand complex, and a scoring function that can rank them, as well as predicting binding affinities by computing, among other values, the Free Energy of Binding (FEB) and the Root Mean Square Deviation (RMSD).

The protein flexibility is a vital issue in docking programs since they perform satisfactorily taking care only the flexibility of ligands. [10, 11]. The methods used for considering the flexibility of ligands in docking experiments cannot be directly assigned to a typical protein due to its vast number of conformational degrees of freedom. Buonfiglio et al. [12] state that ignoring the protein flexibility in docking experiments is indeed a potentially dangerous practice that most likely would result in false-negative outcomes. In fact, proteins are very versatile and their flexibility cannot be a priori neglected since it plays an essential role in their structure and function [12, 13].

To account for the dynamic behavior of proteins, we make use of an ensemble of conformations obtained from a Molecular Dynamics (MD) simulation [14, 15]. MD simulation is one of the most affordable and accurate methods for identifying alternative binding modes of proteins, making possible to understand from fast internal motions to slow conformational changes [14]. The result of an MD simulation is a series of instantaneous conformations, or snapshots, of the protein along the simulation timescale. Throughout this paper, the term Fully-Flexible Receptor (FFR) model [16] is used to refer to the ensemble of snapshots that constitutes an MD trajectory. The major problem in using an ensemble of snapshots during docking experiments is that it becomes a limiting and costly task as the dimensionality of the FFR model increases. Several studies have attempted to deal with this virtual high-throughput screening; however, it remains an unsolved problem [11–13, 17–21].

A number of different methods were proposed in the literature to reduce the elapsed time taken for performing docking-based virtual screening [7]. Most of these methods scale up simulations based on the volume of drug-like compounds by using High-Performance Computing (HPC) environments, such as computing clusters [22, 23], grid computers [24], and cloud computing [25–29]. Despite having different goals and requirements, all these studies carried out in docking small molecules to rigid biological receptors. In ensemble docking experiments, various approaches have been used to reduce the number of MD conformations into a manageable and meaningful set. For instance, some studies have applied clustering algorithms to partition MD trajectories and select only a small set of representative conformations [30–34]. Even though these studies use different functions of similarity to find an optimal clustering, the set of representative MD conformations may interact favorably with some molecules, and unfavorable with others since a small number of structures is used to represent the entire MD trajectory.

A different approach to deal with ensemble docking is addressed by wFReDoW [18], our previous work. This web application was deployed on Amazon Elastic Compute Cloud with the intention of reducing both the overall docking runtime and the dimensionality of a 3.1 ns MD trajectory. wFReDoW reduces the total time of ensemble docking experiments by using a clustering of MD trajectory and identifies partitions with promising snapshots. It claims good results for the experiments presented in [18, 19]. However, the need for information about docking results before submitting a new ligand and the limitation of scalability due to the MPI cluster model are critical aspects of performing molecular docking simulations of FFR models using a large database of small molecules.

In this study, we show that Reduced Fully-Flexible Receptor (RFFR) models can be generated by identifying promising MD conformations to the ligands during the docking experiments without previous assessments about the best free energy of binding or any other evaluation associated with ligand binding quality. To reach this goal, we developed a selective method for optimizing ensemble docking-based experiments for FFR models. This method aims to discard groups of unpromising snapshots for specific ligands at runtime and scale ensemble docking-based experiments on an INhA FFR model out onto cloud virtual machines (VMs). It was deployed on e-FReDock, the cloud-based scientific workflow to perform exhaustive molecular docking simulations of FFR models and multiple ligands [35]. As a result, we expect to significantly reduce the overall execution time of docking experiments and find the best docking poses of the ligands in the resulting RFFR models.

This paper describes the implementation of the proposed method in the e-FReDock workflow [35] and evaluates its results by assessing the quality of the RFFR models produced. It starts with a brief review of the most relevant e-FReDock workflow components and the cloud environments assigned to perform docking experiments on VMs. In the Implementation Section, we detail the novel method developed to select promising MD conformations during docking runtime and introduce the improvements made on e-FReDock to incorporate the selective method. The “Results” section shows the performance of e-FReDock when executed on public VMs and the gains achieved with the proposed method. Such gains were evaluated by analyzing the docking results of the produced RFFR models using AutoDock 4.2 and AutoDock Vina [8]. Furthermore, we also assessed the method gains based on the rigid, crystal structure, of the InhA enzyme. The study ends with a discussion about the findings and future work directions.

## Methods

### The clustered FFR model

The FFR model employed in this study was generated from an MD simulation of the 2-*trans*-enoyl-ACP (CoA) reductase (E.C.1.3.1.9) enzyme or InhA-NADH complex from *Mycobacterium tuberculosis* [36]. InhA is part of the fatty acid biosynthesis system type II (FASII) and plays a role in the synthesis of mycolic acids, which are key components of the *Mycobacterium tuberculosis* cell wall. Inhibition of InhA by the drug isoniazid, for instance, kills the bacteria [36]. The InhA enzyme is one of the best established and validated target for the development of anti-tuberculosis (anti-TB) agents [37, 38].

The MD simulation was performed by the SANDER module from the Amber9 suite of programs [39] using the ff99SB force field [40] by Gargano [41]. According to Gargano [41], the structures belonging to the MD trajectory of the InhA were superimposed onto the initial structure using a rectangular box of 77.7 Å x 73.3 Å x 77.3 Å. Hydrogen atoms, ions, and water molecules were initially submitted to 100 steps of energy minimization with the steepest descent to closely remove contacts of van der Waals forces. The pressure of the simulation was kept at 1 atm and, to avoid disturbance to the system, the temperature was gradually increased from 10 K up to 298 K in six steps (10 K to 50 K, 50 K to 100 K, and so forth). For each step, the velocities were reassigned according to Maxwell-Boltzmann distribution and balanced for 200 ps [41]. Data were saved at every 1 ps over the 20 ns simulation, yielding a total of 20,000 instantaneous receptor conformations. From these 20,000 MD conformations, we discarded the first 500 as being the heating phase of the simulation and use remaining 19,500 as the set of snapshots that constitutes the FFR model of InhA, and it is used to conduct the ensemble docking experiments in this study. Further details on the MD simulations preparation and execution can be found in [41].

To reduce the size of the FFR Model and, consequently, the number of ensemble docking experiments, without affecting the accuracy of the produced RFFR models, we decided to use a clustering of MD conformations as input data for the method proposed. The clustering of MD conformations applied in this study was generated by De Paris et al. [20]. They presented a set of studies to find an optimal partition solution to the 20 ns MD trajectory of the InhA-NADH complex, using structural properties from the substrate-binding cavity of every MD conformation as similarity function for the clustering algorithm. The benefit of using this similarity function for clustering MD trajectories is to have partitions with different patterns of binding modes. For instance, if a receptor conformation belongs to a cluster that interacts favorably with a specific ligand, we can assume that other conformations within the same cluster have similar structural properties in their substrate-binding cavity, and consequently, will behave similarly. Otherwise, if the interaction between the same receptor and ligand is unfavorable, we can consider that this cluster has unpromising snapshots and can be discarded to reduce the number of docking experiments on the FFR model [42]. Due to this high level of binding cavity similarity within a cluster, we used the optimal clustering solution selected by De Paris et al. [20] as input to the method proposed in this study.

### e-FReDock: The flexible receptor docking-based virtual screening workflow

The e-FReDock workflow was developed in e-Science Central (e-SC) [43], a workflow enactment system for the development of portable analytics applications that can be deployed on dedicated hardware or in a cloud-based environment. A typical workflow in e-SC is composed of blocks of activities (or services) to orchestrate the execution flows based on a direct acyclic graph representation.

The previous specification of e-FReDock deployed on e-SC is presented in De Paris et al. [35]. It was designed on cloud-based environments and contains two sub-workflows: Create Experiment, which creates new docking experiments of an FFR model and one ligand; and Ensemble Docking Experiment, which includes a set of blocks for performing molecular docking simulations on AutoDock4.2 [8] by scaling each sub-workflow out onto Azure VMs. The e-FReDock workflow also stores essential docking information on MongoDB [44].

The e-FReDock workflow uses the e-SC API Java client to control the invocations of both sub-workflows. This API has a set of e-SC components to execute workflow instances on cloud resources and manage data files by accessing the e-SC file system. We decided to use this API to deal with the quality assessment of the groups of snapshots at docking runtime since the e-SC enactment system is a directed acyclic graph based workflow, i.e., it can not repeat workflow tasks. Thus, besides creating new blocks of activities to meet the needs of the proposed method, we also performed some changes in the e-SC API to monitor the selective ensemble docking-based experiments.

### Cloud computing platforms

The cloud platforms selected for performing the ensemble docking-based experiments in this study were: Microsoft Azure public cloud [45] and Cloud Innovation Centre (CIC) private cloud [46]. Azure was chosen for this study since it is one of the most well-known and well-established cloud platforms. Some studies have used Azure cloud instances to optimize the RDD process, such as prediction of chemical activity using e-SC [47] and virtual screening practices [25, 28].

The second cloud platform used to execute our experiments was CIC. This private cloud is located at Newcastle University (UK) and built by the School of Computing Science to support cloud research, staff and students’ mass-scale virtualization requirements and third-party partners. CIC private cloud infrastructure is a virtualization platform, consisting of 27 nodes with 20 cores each, resulting in a total of 540 cores and 7424 GB total RAM. The storage area network uses a 10 Gb Ethernet LAN and 4 nodes with 12 cores, 64 GB RAM and 37 TB storage per node. Furthermore, 3 nodes with 12 cores, 64 GB RAM and 1.4 TB storage each are used for management purposes. Horizon Dashboard [46] is the web-based user interface for OpenStack Nova services. Its access was granted by the project coordinators for the sole purpose of running the experiments of this research.

## Implementation

### The selective approach for optimizing ensemble docking-based experiments

**Fig. 1:**
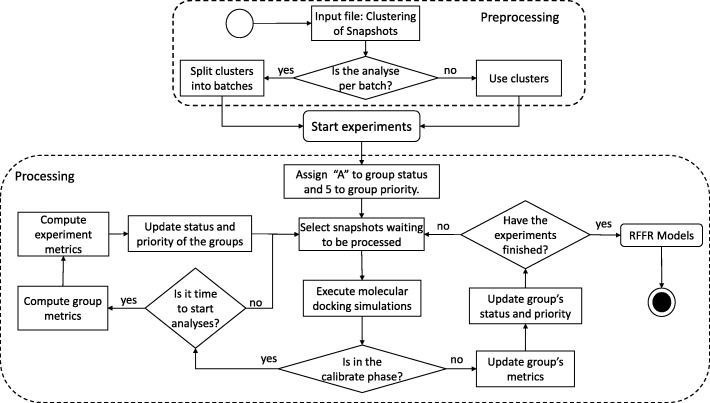
Strategic method for performing the selective method for optimizing ensemble docking-based experiments in one ligand. Calibration phase is the process of quantitatively defining interactions between a sample of MD conformations and a ligand

The selective approach aims to identify and discard snapshots with unfavorable receptor-ligand bound conformations in groups of MD conformations with similar properties in their substrate-binding cavities. Favorable binding modes are discovered and ranked during the docking experiments, based on predicted FEB values extracted from snapshots already docked. The approach developed to perform selective ensemble docking experiments is divided into preprocessing and processing stages. The schematic process from these both stages is given in the flowchart shown in Fig. 1.

An experiment is created when a clustering of MD Conformations and a ligand are submitted as input for docking executions. Before starting the experiment, the user should define the percentage and the number of minimum and maximum snapshots per batch. Based on these parameters, the preprocessing phase splits clusters of snapshots into batches. Even though the proposed method allows to choose a type of analysis, we performed evaluations for both, batch and cluster, and concluded that performing analyses in small samples of snapshots (batch analyses) identifies more precisely promising snapshots than in cluster analyses. For this reason, all results presented in this study were performed by using analyses per batch.

**Fig. 2:**
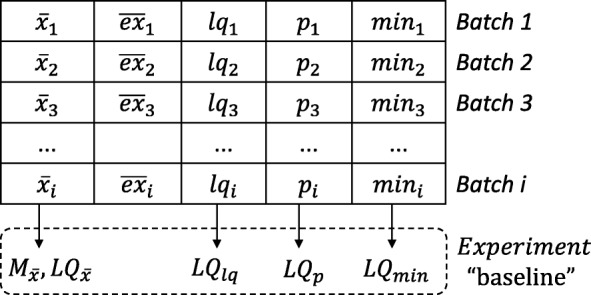
Schematic representation of the metrics used for computing the experiment baseline. The metrics of the experiment baseline are based on the FEB values computed for each batch, where median and lower quartile are taken from $\overline {x}_{i}$, and lower quartiles from the *lq*_*i*_, *p*_*i*_ and *min*_*i*_

Each batch contains its status and priority, used for determining the order in which the snapshots will be processed. Priority indicates how promising a group of snapshots is on a scale from 0 to 5 (5 being the most promising), whereas status denotes one of the following four possibilities: (A) Active, (C) Calibrate phase, (D) Discarded and (F) Finished. In this approach, when a docking experiment is submitted to be executed, all batches receive status “A” and priority 5. Snapshots are processed until the percentage threshold to start the analysis, which is a parameter defined by the user, is reached by all batches of an experiment. The highest priority is set to accelerate the end of the calibrate phase. When all batches reach the percentage threshold to start the analysis (i.e., all batch with status assigned to “C”), their statuses are simultaneously changed to “A”, and a set of metrics are computed to define the experiment baseline. Figure 2 shows the metrics used to compute the experiment baseline from the snapshots processed in the Calibrate phase.

The set of metrics computed after the calibrate phase are sampling FEB average ($\overline {x}_{i}$), estimated FEB average ($\overline {ex}_{i}$), sampling FEB lower quartile (*lq*_*i*_), sampling FEB 13^*th*^ percentile (*p*_*i*_), and sampling FEB minimum value (*min*_*i*_). The estimated FEB average is defined by Hübler et al. [48] as

1 $$ \overline{ex}_{i} = \frac{1}{n_{i}} \left(\sum_{x\epsilon B_{i}}x + (0.4985 \times r_{i} \times (2\overline{x}_{i} - s_{i}))\right)  $$

and

2 $$ {s}_{i} = \sqrt{\frac{1}{n_{i}-1} \left(\sum_{x\epsilon B_{i}}(x - \overline{x}_{i}\right)^{2}}  $$

where *n*_*i*_ is the number of snapshots in batch *i*, *r*_*i*_ is the number of remaining snapshots to be processed from batch *B*_*i*_, *x* is the best predicted FEB value for each snapshot from batch *B*_*i*_, and $\overline {x}_{i}$ is the sampling average. Figure 2 shows how the method computes the set of metrics where rows represent the values from each batch and columns represents the values used to define the experiment baseline metrics.

After the calibrate phase, our method selects batches of snapshots with status equal to “A” and uses the priority to dictate the order in which the snapshots are processed. The higher the priority of a batch, the greater the amount of its snapshots are selected and processed. An experiment ends when all batches hold status equal to “D” or “F”. Promising snapshots are those belong to batches that process all snapshots (Status “F”). A batch with the status equal to “D” is stopped as it contains snapshots with poor quality of docking results for a specific ligand. A batch may be discarded for two reasons: (i) if it is unable to reach the experiment baseline metrics (see Fig. 2) or; (ii) if it has low priority and reaches the percentage threshold to discard a batch, which is also defined by the user.

In the analyses of docking results, the desirable batches (i.e. batches with priority 5 are those where: (a) $\overline {x}_{i}$ and $\overline {ex}_{i}$ are less or equal to ${LQ}_{\bar {x}}$; (b) *lq*_*i*_ is less or equal to *LQ*_*lq*_; (c) *p*_*i*_ is less or equal to *LQ*_*p*_; and (d) *min*_*i*_ is less or equal to *LQ*_*min*_. If a batch does not meet such conditions, its priority is decreased, tending to zero when $\overline {x}_{i}$ and $\overline {ex}_{i}$ are higher than $M_{\bar {x}}$. We have computed the lower quartiles, the 13^*th*^ percentile, and sampling minimum values since we expect to outperform the quality of the RFFR models produced not only by considering the FEB values average but also by identifying the snapshots that account for at least 25% more negative FEB values of a batch.

### The advances on e-FReDock workflow for handling the selective ensemble docking-based method

**Fig. 3:**
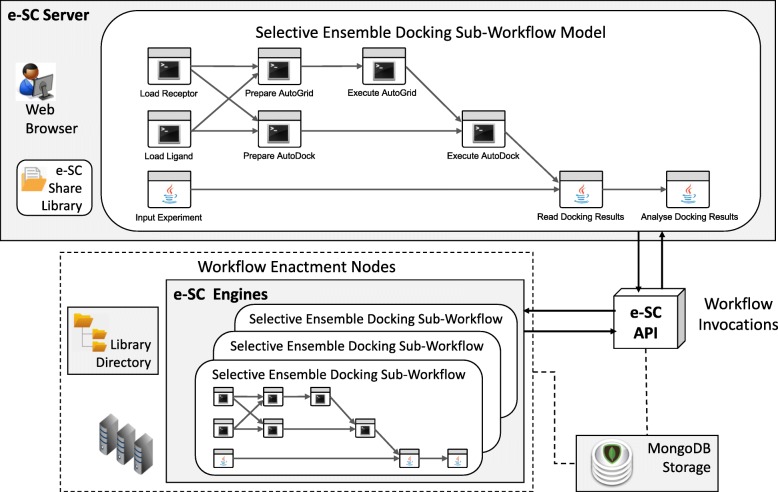
The Selective Ensemble Docking Sub-Workflow from e-FReDock based on e-SC. The e-SC Server contains the workflow model, which is sent to be executed on one of the enactment nodes. The bottom box represents the pool of virtual machines attached to the e-SC server from which workflow instances are executed

The primary objective of introducing the proposed method into the e-FReDock scientific workflow was to assist in performing practical virtual screening on FFR models by speeding up ensemble docking experiments. Towards this end, we made improvements and refinements in the original e-FReDock workflow version by the approach described in the previous section. Figure 3 shows the selective ensemble docking sub-workflow along with the native operations of e-FReDock on e-SC. To include the selective approach proposed in this study, we created a new block in the selective ensemble docking sub-workflow and a set of functions in the e-SC API.

The Analyze Docking Result block, which was added in the selective ensemble docking sub-workflow, computes the priority and determines the status of each group of snapshots by using the set of metrics described in the previous section. Priorities, status and other data necessary for handling the proposed method are stored in the MongoDB database, which in turn, is also accessed by the e-SC API for discarding groups of unpromising snapshots. The e-SC API is one of the essential components of the e-FReDock conceptual architecture and it is based on the workflow scheme from Fig. 1. It contains every procedure required to scale the selective ensemble docking sub-workflow out onto VMs, monitors the Selective Ensemble Docking sub-workflow invocations, and selects snapshots that are likely to represent the most promising conformations between the FFR model and a specific ligand. Data and control flows are monitored by e-SC, which is also responsible for scaling VMs onto cloud platforms.

## Results

### e-FReDock performance analyses on Azure virtual machines

To better understand which choices to make regarding costs and performance of a commercial cloud system, we performed and evaluated a set of experiments on e-FReDock, using Azure Dv2-series instances located in the North Europe data center. docking The Dv2-series Ubuntu 14.04 instances are based on the 2.4 GHz Intel Xeon E5-2673 v3 processor with Intel Turbo Boost Technology 2.0 that can go up to 3.2 GHz. Table 1 lists the different VMs instances we tested along which their corresponding features and costs.

**Table 1:** Types of Azure Dv2-series instances used to assess e-FReDock performance

Instance name	Cores	RAM (GB)	Disk size (GB)	Price (US$)^a^
D2 v2	2	7	100	0.14
D3 v2	4	14	200	0.28
D4 v2	8	28	400	0.55
D5 v2	16	56	800	1.11
D11 v2	2	14	100	0.18
D12 v2	4	28	200	0.37
D13 v2	8	56	400	0.74
D14 v2	16	112	800	1.48

^a^Pricing information from the Azure website as of January 15, 2016 [45]

In these experiments, the Lamarckian Genetic Algorithm (LGA) from AutoDock4.2 and its parameters were used to execute the molecular docking simulations between snapshots from the InhA FFR model [41] and the TCL ligand from PDB ID 2B35 [49] with 2 rotatable bonds. Twenty-five LGA independent runs were executed with a maximum of 500,000 energy evaluations. The e-SC server and MongoDB were hosted in a Standard D2 VM instance (Intel Xeon 2.4 GHz, 7 GB RAM). A total of 100 Selective Ensemble Docking sub-workflow invocations were executed in Dv2-series machines with different workloads to identify a setting that makes more efficient the use of available resources. For this purpose, we evaluated the efficiency regarding speedup per processor with the intention of measuring how many tasks can be executed in parallel to avoid wasting resources.

**Fig. 4:**
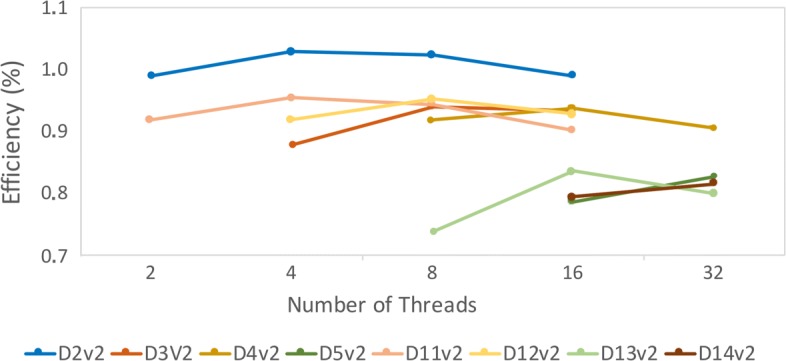
Comparing the efficiency of Dv2-2 Azure instances with different number of threads

As can be seen in Fig. 4, virtual machines with smaller number of cores presented better efficiency than bigger ones. Another interesting finding is the high efficiency observed in instances with small RAM and an equal number of cores. It suggests that the amount of RAM does not affect the docking experiments efficiency, regardless of the number of threads. As the RAM is a key aspect of the instance price and considering our performance e-FReDock tests, we decided to run the cost-effectiveness analyzes on instances with small RAM sizes.

**Fig. 5:**
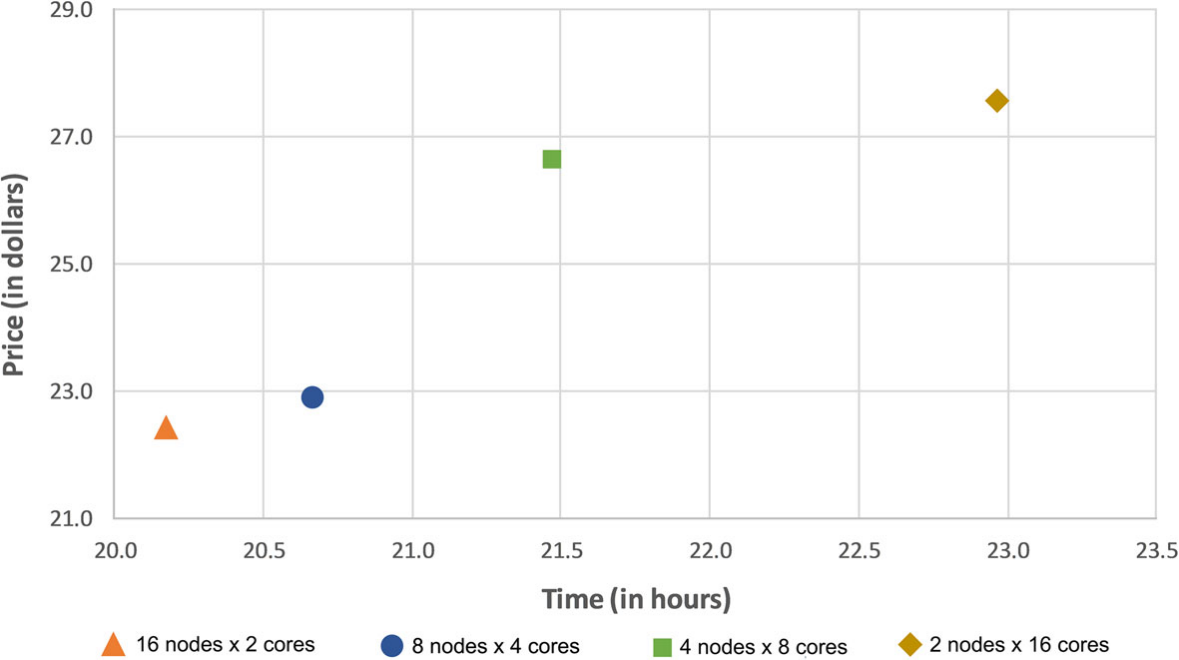
Performance analysis on Azure VM. The Azure instances used are D2 v2, D3 v2, D4 v2 and D5 v2 with 2, 4, 8 and 16 cores, respectively. Pricing and instance information from the Azure website as of January 15, 2016

The Fig. 5 shows the estimated elapsed time and costs to execute simultaneously 32 docking experiments in the D2-series Azure instances. The estimation was determined on 19,500 Selective Ensemble Docking sub-workflow invocations, which is the number of snapshots from the clustered FFR model. Interestingly, the time spent to execute docking experiments increases as the number of cores per instance rises. This observation suggests that AutoDock4.2 is unable to manage multiple LGA (i.e., more than 4) in the same machine since its efficiency is affected by the workload. Thus, we decided to execute the e-FReDock workflow in a pool of D2 v2 Azure instances.

It is worth emphasizing that LGA is a non-deterministic algorithm and its overall time execution may vary according to the global search space of genetic algorithms. This search randomly generates a population of ligand poses until either the maximum number of evaluations or the maximum number of generations limits is reached [8]. As the population is generated randomly, the genetic algorithm may not present the same behavior, even for the same input. For this reason, Fig. 4 shows the efficiency of D2v2 instance larger than 1. However, we monitored the resource use on Azure portal when a set of 10 VMs was running the experiments, and the average percentage of CPU use was 98%. It indicates the good efficiency of the VMs even when more than one virtual machines are simultaneously used to run many tasks of LGA algorithm.

### Analysis of the e-FReDock results

#### e-FReDock configuration protocol

To execute the selective ensemble docking-based experiments on e-FreDock, we select a set of 74 ligands from two databases: 12 from PDB [50] and 62 from ZINC [51]. The selection approach used to select ligands from PDB was to discard structures that are mutant or without NADH or complexed with coenzyme NADH as an adduct. The latter structures were unselected as the 1ENY structure - the crystallography structure of the FFR model - is already complexed with the NADH coenzyme. We also discarded those structures that contain the substrate analog (THT) or more than one ligand within the substrate-binding cavity. As ZINC database [51] is the second biggest repository of small compounds ready to execute in docking software, we employed the ZINCPharmer online interface [52] to construct and refine the pharmacophore models based on the most effective anti-TB drugs: rifampicin and isoniazid [53]. A set of pharmacophore properties were extracted from these two ligands and were used as restrictions to ZINCPharmer search for new ligands in ZINC database. The result of this investigation was a list of 957 ligands, which in turn were sorted by the minimum predicted FEB values obtained by performing docking experiments with a small set of 25 representative structures of the FFR model [54]. The first 62 compounds from this list of ranked compounds were selected to conduct our experiments.

Docking parameters were set up to perform 20 LGA independent runs with a maximum of 500,000 energy evaluation. The grid box was centered in the middle coordinates of the binding cavity with a dimension of 48Å X 48Å X 44Å for ZINC’s compounds, and customized sizes were configured to the PDB’s ligands. All ligands were treated as flexible during the docking experiments. To provide the reference pose of each PDB ligand, we first fit all snapshots of the FFR model to the first MD conformation. After that, we placed the reference pose of each PDB ligand based on the first MD conformation and reproduced it for all MD conformations. A PDBQT file for each snapshot from the FFR model was created before starting the experiments and placed into the e-SC Share Library. We set the atom types used by AutoDock4.2, added the Kollman charges and merged all receptor snapshots from the FFR model with the nonpolar hydrogens. For each experiment, groups were divided into batches of 20%, limiting the number of snapshots between 50 and 150. The percentages of processed snapshots defined to start the analyses and to discard a batch were 10 and 40%, respectively. These values were obtained based on preliminary test analises.

The e-FReDock experiments were performed on the two cloud environments: CIC [46] and Microsoft Azure. Each cloud environment was configured to have its e-SC server. The e-FReDock setup consists of installing and configuring e-SC system and MongoDB into the e-SC server. The same e-SC server used to perform the performance analysis on Azure instances was employed to perform these experiments. Blob storage with 30 GB was allocated to deploy the e-SC server on Azure, and a hard disk with 40 GB was attached to the e-SC server on the CIC private cloud. Based on the performance analyses described in the last Section, we decided to attach 10 D2 v2 Azure VMs into the e-SC server, where each VM was set to run 4 parallel workflow invocations (4 threads). CIC private cloud has a small set of flavors with a limited hard disk. Disk size was the determining factor to select the VM flavors since the Ubuntu 14.04.3 LTS installation takes 7.5 GB of the total disk size. For this reason, the 10 biggest CIC instances, each one with 4 cores, 8 Gb RAM, and 16GB disk size, were selected to deploy e-FReDock in a pool of private VMs.

#### Evaluating the accuracy of the RFFR models

The method proposed in this study aims to eliminate groups of unpromising snapshots at docking runtime using the approach to perform selective ensemble docking experiments presented in the Implementation Section. This method generates an RFFR model for each ligand based on a set of metrics computed to assign the priority and status for each batch. To validate the e-FReDock results, we statistically compared the set of snapshots that constitutes the RFFR model with a set of snapshots selected by chance from the ensemble docking experiment. Thus, the following hypotheses are addressed: (i) Null Hypothesis (*H*_0_): the method does not result in gains; (ii) Alternative Hypothesis (*H*_1_): the method results in gains. To reject the null hypothesis, the accuracy of all RFFR models produced should be higher than the selective ensemble docking at random, considering the same percentage of processed snapshots.

The quality of the RFFR models produced by e-FReDock was analyzed by scoring the number of snapshots that are in the top 10, 20, 30, 100 and 200 best ensemble docking results of the whole FFR model for each ligand. Tables 2 and 3 report the performance of the RFFR models produced after executing e-FReDock. The most striking result to emerge from generated RFFR models is the high accuracy reached by ZINC ligands, with top best FEB cases ranging, on average, from 89 - 94% and the model size reduced by approximately 57% (see Table 3). Furthermore, e-FReDock was able to cover all the best 10, 20 and 30 interactions in 47% (29), 29% (18) and 18% (11) of the 62 ZINC ligands, respectively.

**Table 2:** Accuracy assessments in the e-FReDock scientific workflow for InhA’s known inhibitors

PDB ID	Ligand	Proc. Snap. (%)	TOP10 (%)	TOP20 (%)	TOP30 (%)	TOP100 (%)	TOP200 (%)
1P44	GEQ	48.92	100.00	95.00	93.00	86.00	86.00
2B35	TCL	50.67	80.00	55.00	60.00	75.00	80.00
2B36	5PP	50.97	60.00	70.00	66.00	67.00	73.00
2B37	8PS	49.72	60.00	60.00	60.00	78.00	79.50
2H7I	566	52.33	80.00	90.00	83.00	79.00	78.00
2H7L	665	50.62	100.00	100.00	100.00	98.00	98.00
2H7M	641	47.75	100.00	100.00	100.00	100.00	100.00
2H7N	744	50.65	100.00	100.00	100.00	97.00	97.00
2H7P	468	49.84	90.00	95.00	83.00	82.00	82.00
3FNE	8PC	52.46	90.00	90.00	93.00	90.00	88.00
3FNH	JPJ	47.89	100.00	100.00	100.00	96.00	93.00
2NSD	4PI	53.61	100.00	95.00	96.00	97.00	96.00
Average	-	50.45	88.83	87.50	86.17	87.08	87.54

**Table 3:** Accuracy assessments in the e-FReDock scientific workflow for ZINC chemical compounds

Ligand	Proc. Snap. (%)	TOP10 (%)	TOP20 (%)	TOP30 (%)	TOP100 (%)	TOP200 (%)
91870997	47.23	90.00	90.00	93.00	90.00	86.00
35361468	43.79	100.00	100.00	100.00	99.00	93.50
63349859	44.25	100.00	95.00	93.33	93.00	90.00
12047789	42.64	100.00	100.00	100.00	92.00	88.00
56919632	46.33	100.00	100.00	93.00	85.00	84.00
63479951	35.79	90.00	80.00	80.00	80.00	81.50
53364786	41.11	80.00	75.00	76.67	90.00	89.50
6144048	46.06	100.00	95.00	96.67	96.00	96.00
39532319	47.03	90.00	90.00	90.00	88.00	85.00
34378053	45.02	90.00	95.00	96.67	94.00	92.50
41584161	41.86	90.00	95.00	93.33	89.00	84.00
41584148	42.37	90.00	85.00	86.67	88.00	87.00
1456628	45.20	100.00	95.00	96.67	90.00	89.50
36676865	45.33	100.00	95.00	93.33	95.00	95.00
90914428	43.37	90.00	85.00	86.67	80.00	84.50
63503064	42.09	100.00	100.00	100.00	96.00	93.50
17243209	39.96	90.00	90.00	93.33	95.00	94.50
41584175	57.70	90.00	90.00	90.00	86.00	89.50
23360796	41.48	90.00	85.00	83.33	90.00	87.00
65298323	35.53	100.00	85.00	83.33	80.00	75.00
34378052	42.18	90.00	95.00	96.67	95.00	93.00
9251152	38.69	90.00	95.00	96.67	96.00	95.00
11871395	43.82	100.00	100.00	100.00	98.00	90.50
9197776	43.89	80.00	80.00	73.33	75.00	78.00
90185596	45.59	100.00	85.00	86.67	91.00	90.50
9197790	42.19	90.00	90.00	90.00	74.00	74.00
24000894	42.76	100.00	100.00	100.00	99.00	98.50
39923320	42.05	90.00	95.00	96.67	98.00	98.00
64625806	44.73	80.00	85.00	86.67	90.00	92.50
64040549	44.18	100.00	100.00	100.00	99.00	99.00
64057877	45.66	90.00	95.00	96.67	98.00	97.00
9130690	45.92	100.00	100.00	100.00	98.00	96.00
63479935	35.05	100.00	100.00	96.67	91.00	90.50
63362881	38.35	100.00	90.00	93.33	89.00	87.00
38570167	47.10	90.00	90.00	86.67	87.00	86.00
64074412	46.46	90.00	95.00	93.33	97.00	94.00
64002358	41.70	90.00	95.00	93.33	96.00	94.50
4335232	44.70	80.00	90.00	90.00	88.00	87.50
72047160	46.02	100.00	100.00	93.33	93.00	96.00
9409766	40.57	100.00	100.00	96.67	92.00	92.50
64103060	46.31	100.00	95.00	96.67	92.00	91.00
89608939	43.58	100.00	90.00	86.67	87.00	83.50
2911927	39.10	90.00	90.00	80.00	80.00	79.00
41584155	44.88	90.00	90.00	90.00	94.00	90.50
8323837	43.53	90.00	95.00	96.67	97.00	96.50
64074451	46.88	90.00	85.00	76.67	91.00	88.00
64889693	43.07	90.00	75.00	76.67	76.00	77.00
20285686	40.80	100.00	100.00	100.00	89.00	88.00
15038988	45.65	100.00	100.00	100.00	98.00	97.50
9522091	45.55	90.00	95.00	96.67	94.00	95.50
20836860	45.79	90.00	95.00	96.67	89.00	90.00
6648224	44.68	100.00	100.00	96.67	96.00	94.00
35727540	42.32	100.00	100.00	96.67	96.00	95.50
65298175	39.48	80.00	75.00	83.33	74.00	73.50
11074320	40.34	100.00	100.00	96.67	96.00	96.00
9197821	42.35	90.00	85.00	80.00	73.00	77.00
2924572	39.86	70.00	85.00	86.67	86.00	83.00
8971422	45.14	100.00	100.00	100.00	96.00	95.50
25286217	47.10	100.00	90.00	90.00	89.00	86.50
5200961	39.55	90.00	95.00	90.00	88.00	86.50
14989185	46.13	100.00	100.00	100.00	95.00	95.00
2347739	39.88	100.00	95.00	96.67	91.00	90.50
Average	43.35	93.55	92.58	92.03	90.44	89.44

Even though the RFFR models generated by PDB ligands showed lower quality than those produced by ZINC chemical compounds (on average between 86 and 89%), the worst results were obtained only on 3 structures (2B35_TCL, 2B36_5PP, and 2B37_8PS). These findings suggest that MD conformations from the FFR model used in this study are unable to reproduce structures with tight-binding InhA inhibitors and with sub-nanomolar affinities, i.e. structures that have very similar mode of action to triclosan [49].

The analyses on e-FReDock results provide support to reject the null-hypothesis defined as *“the method does not result in gains"*. A random selection of 9837 snapshots - equivalent to 50.45% of processed snapshots for PDB ligands - and 8453 - equivalent to 43.35% of processed snapshots for ZINC compounds - would statistically take around 43.00 to 50.00% of the best 10, 20, 30, 100 and 200 receptor-ligand interactions. Tables 2 and 3 demonstrate that the lowest percentage of the top snapshots selections was 55% for the 20 best interactions between the FFR model and 2B35_TCL ligand. Nevertheless, this percentage is still higher than the processed snapshots, i.e., 50.67%. Furthermore, the percentage reached by the 2B35_TCL ligand in the others top best FEB cases are higher or equal to 60%.

**Fig. 6:**
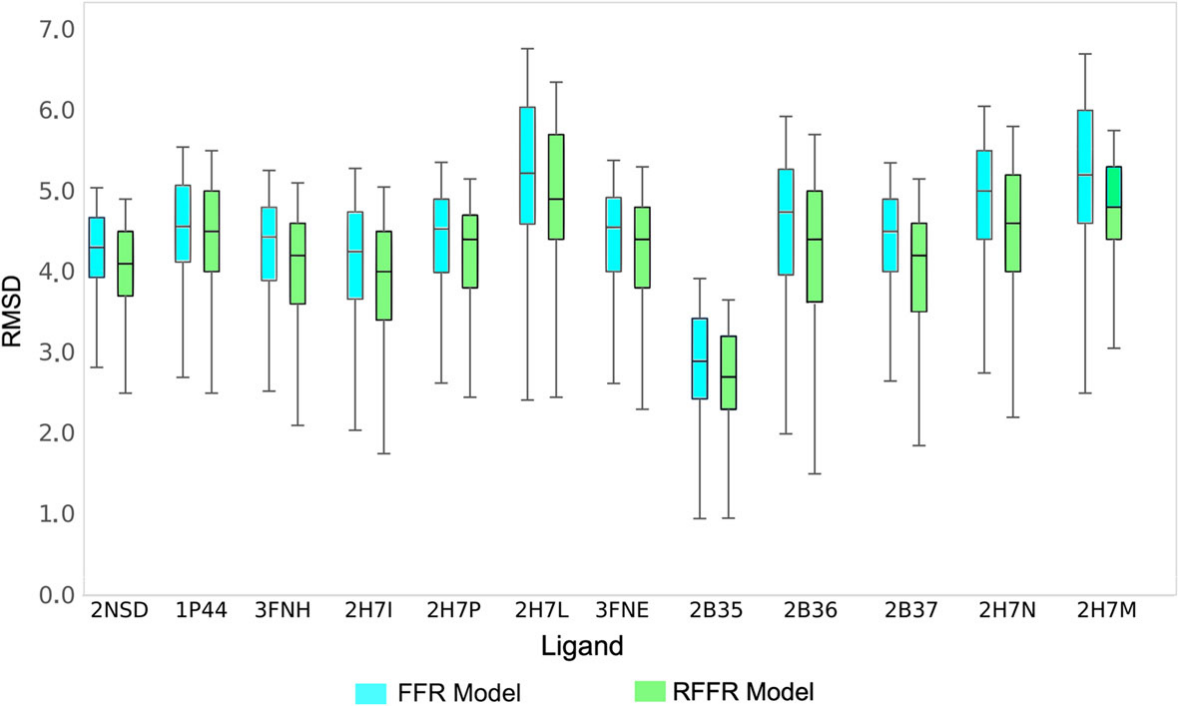
Comparison between the RMSD values obtained by the FFR model and the resulting RFFR models for the InhA’s known ligands. Boxplots represent the trends in RMSD values changes per ligand. Values range from the first quartile to the third with the median RMSD values denoted by the black line across the central box region

To further validate the gains of the proposed method, the alternative hypothesis, we also assessed the RMSD values of the RFFR models produced for ligands extracted from PDB. The goal of this analysis is to investigate if, in addition to cover the best interactions, e-FReDock is also able to select the best RMSD values. For that, a comparative analysis of the variation of RMSD values between the FFR model and the RFFR models is presented in Fig. 6. It is noticeable that boxplots from the RFFR models report central tendencies lower than those presented by boxplots from the FFR models. RFFR models also present the minimum observation values (lower whiskers) lower in almost all cases. Therefore, it can be stated that e-FReDock was also able to cover snapshots with the lowest docking final poses for almost all ligands, even though the method proposed in this study is based only on FEB values.

Regarding docking accuracy, Fig. 6 shows that TCL (PDB ID: 2B35) ligand is close to its reference poses, while the remaining ligands have RMSD values not lower than 2,00 Å. This RMSD threshold value is used along with the predict FEB value for selecting satisfactory docking results [8]. We have performed a more detailed study on the 20 ns MD trajectory of the InhA-NADH complex to identify new InhA inhibitors based on its substrate-binding cavity, which ranges from 45.4 Å^3^ to 2,852.9Å^3^ for the entire 20 ns MD trajectory [20]. Hence, ligands with smaller atom counts and molecular weights are more likely to interact with one of the MD conformations. For instance, Fig. 6 shows that TCL (PDB ID: 2B35) ligand have the best RMSD values and its molecular weight is 289.54 g/mol and atom count is 24. Other ligands present higher values of both, molecular weights and atom count.

#### Comparing docking results between RFFR models and the 1ENY crystallographic structure

In this set of experiments, we intend to evaluate the quality of the RFFR models produced based on the assumption that our selective method was able to outperform docking results when compared with the rigid structure that originated the FFR model (1ENY Crystallographic Structure [36]). Towards this end, FEB values obtained from docking experiments were the measure selected for evaluating interactions between MD conformations and different ligands. To evaluate the gains and losses obtained by exploring the explicit flexibility of receptors in the selective method proposed, we compute the accuracy of docking results obtained between RFFR models, which were produced in the e-FReDock workflow, and the 1ENY structure for the same set of ligands. To identify which structure (flexible or rigid) reached the best docking results, we classified each tested ligand into one of the following categories:

1. RFFR model winner: If the best predicted FEB value reached by the RFFR model outperforms the best predicted FEB value from the 1ENY structure in more than 1 kcal/mol.
2. Tie for RFFR model: If the best predicted FEB value reached by the RFFR model outperforms the best predicted FEB value from the 1ENY structure with a difference equal to or less than 1 kcal/mol.
3. Tie for 1ENY structure: If the best predicted FEB value reached by the 1ENY structure outperforms the best predicted FEB value from the RFFR model with a difference equal to or less than 1 kcal/mol.
4. 1ENY structure winner: If the best predicted FEB value reached by the 1ENY structure outperforms the best predicted FEB value from the RFFR model in more than 1 kcal/mol.

The rate threshold of 1 kcal/mol used to identify the winners is based on the approach used by Huey et al. [55]. They validated the accuracy of docking experiments and concluded that AutoDock4.2 was able to satisfactorily predict the binding affinities for about 80% of docking results when the final poses and the FEB values vary up to 2.5 Å and 1 kcal/mol from the crystal structure.

The best FEB values obtained by ligands for the 1ENY crystallographic structure and the RFFR model were assessed and ranked (see Additional file 1: Table S1). Table 4 summarizes the winners for ZINC and PDB ligands according to the four categories employed described above. It is clear to see that the resulting RFFR models were able to outperform the 1ENY crystallographic structure for all ligands. This finding was unexpected and corroborates with the evidence that receptor flexibility provides significant docking improvements compared with a rigid treatment of the protein [12, 21].

**Table 4:** Comparative analysis of the docking results obtained from the RFFR models produced by e-FReDock and the rigid-protein

Database	Ligands	1ENY Winners	RFFR Winners	Ties for 1ENY	Ties for RFFR
PDB	12	0.00%	16.67%	25.00%	58.33%
ZINC	62	0.00%	85.48%	0.00%	14.52%
Total	74	0.00%	74.32%	4.00%	21.62%

Another interesting finding was that 83.33% of selected PDB ligands (25% tie for 1ENY and 58.33% tie for RFFR) presented FEB values very close to the 1ENY structure, while the RFFR model outperforms its rigid model for only 2 ligands (16.67%). These results may be related to the fact that InhA structures from PDB are unable to be reproduced by any MD conformation of the FFR model [20]. Conversely, the considerable number of RFFR winners for ZINC ligands (85.48%) reveals that MD conformations from the FFR model have new binding modes to be explored, resulting in a set of ligands with satisfactory FEB values and significant difference regarding the 1ENY crystallographic structure.

### Assessments in the selective method simulation using AutoDock Vina

This set of experiments was performed to investigate the performance of the proposed selective method when it is applied to a different docking method. We decided to use AutoDock Vina [8] since it is also freely available and widely used by the academic community, but its scoring function addresses a different requirement from that used in AutoDock4.2 [9]. To evaluate our method, first we performed docking experiments between the FFR model and 12 different ligands (4 from PDB and 8 from ZINC database) using AutoDock Vina. After that, we extracted the docking results to simulate the selective docking-based virtual screening method. The maximum number of binding modes was set to 20 and the exhaustiveness of search was set to 4. Remaining parameters were maintained unchanged. The preparation protocol of the snapshot receptors and ligands, and the reference parameters of the selective method were the same to those used in e-FReDock. Results are showed in Table 5.

**Table 5:** Performance of the selective docking-based virtual screening method using AutoDock Vina evaluated against 12 different ligands, obtained from PDB and ZINC databases

PDB ID	Ligand	Proc. Snap. (%)	TOP10 (%)	TOP20 (%)	TOP30 (%)	TOP100 (%)	TOP200 (%)
2B36	5PP	45.00	100.00	100.00	96.00	91.00	94.00
2B37	8PS	53.00	100.00	95.00	96.00	96.00	95.00
2H7M	641	54.00	90.00	90.00	93.00	88.00	88.00
2B35	TCL	64.00	80.00	90.00	83.00	88.00	88.00
-	53364786	68.00	100.00	95.00	96.00	97.00	95.00
-	34378053	52.00	100.00	95.00	96.00	94.00	93.00
-	39923320	64.00	70.00	70.00	76.00	81.00	82.00
-	63479935	53.00	80.00	85.00	76.00	59.00	54.00
-	64074412	61.00	90.00	80.00	80.00	79.00	79.00
-	89608939	49.00	70.00	60.00	56.00	60.00	59.00
-	9522091	49.00	100.00	95.00	96.00	89.00	84.00
-	20836860	64.00	100.00	90.00	93.00	94.00	94.00
Average	-	56.33	90.00	87.08	86.42	84.67	83.58

As we expected, our method is able to reduce the number of processed snapshots on average by 56.33% in AutoDock Vina, while keeping the quality of the resulting RFFR models. The positive accuracy is evidenced by the percentage of the best docking results selected for each ligand, which ranges from 54.00 to 100.00% with an average superior to 83.00% for all top best results analyzed. These results provide further support for the alternative hypothesis (*H*_1_) defined to validate the e-FReDock results, which is accepted when our selective method results in gains by comparing statistically to a random selection. Even though some ligands present the percentage of processed snapshots higher than 50%, the minimum percentage of top best docking results was 54.00%. According to this finding, we can infer that the selective docking-based virtual screening method proposed in this study may be applicable to different docking methods.

**Fig. 7:**
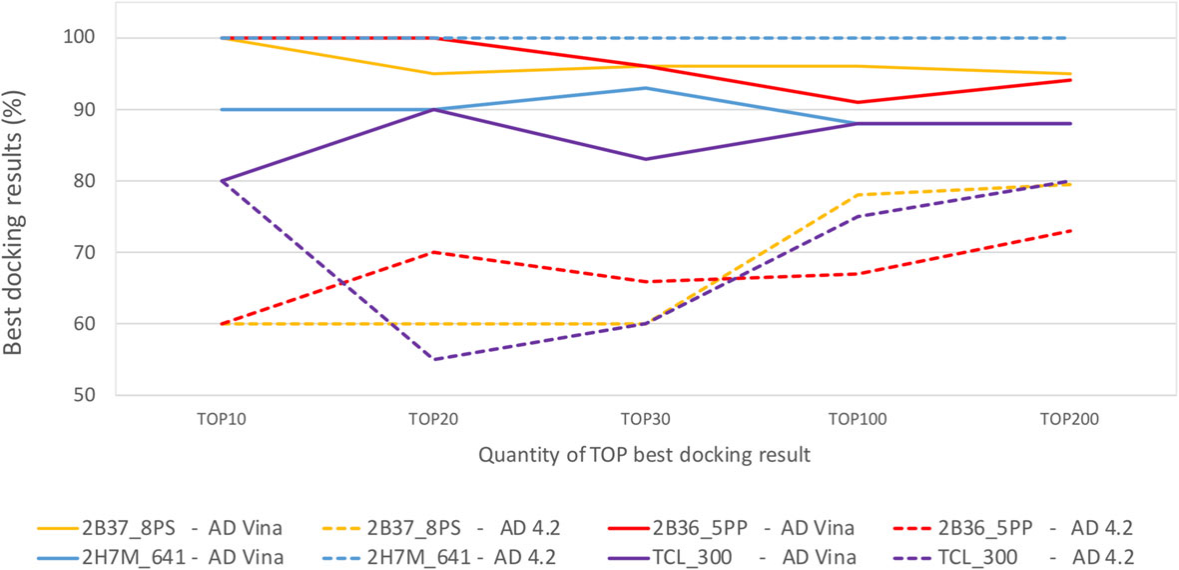
Comparative analysis on the accuracy of PDB ligands for the selective method using AutoDock4.2 and AutoDock Vina. Data from AutoDock4.2 and AutoDock Vina were extracted from Tables 2 and 5, respectively

**Fig. 8:**
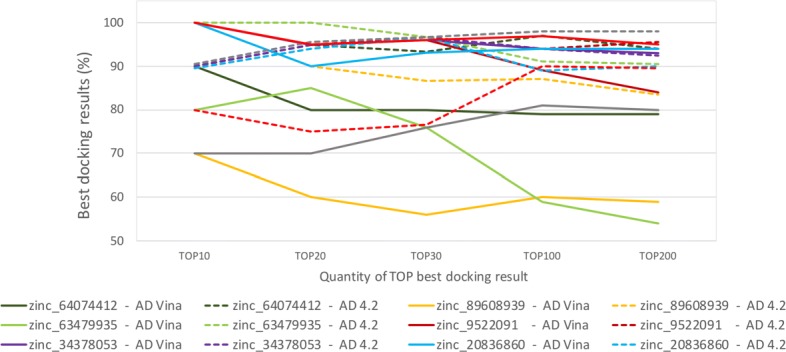
Comparative analysis on the accuracy of ZINC ligands for the selective method using AutoDock4.2 and AutoDock Vina. Data from AutoDock4.2 and AutoDock Vina were extracted from Tables 3 and 5, respectively

Overall, AutoDock Vina processed more snapshots than AutoDock4.2. The average percentage of processed snapshots using AutoDock Vina was 54.00% for PDB ligands and 57.50% for ZINC ligands, whereas using AutoDock4.2 for the same ligands was 49.78 and 43.07% from PDB and ZINC databases, respectively. As can be seen in Figs. 7 and 8, the accuracy does not always improve in the same proportion that the percentage of processed snapshots increases. For instance, ligands from ZINC database processed more snapshots using AutoDock Vina and their performance were equal or less than those reached by AutoDock4.2. This finding suggests that the proposed selective method may be used for different docking methods, but the accuracy depends on the structure under study and the scoring function used to identify the most promising snapshots of FFR models.

## Discussion

The selective method proposed in this study aims at identifying promising MD conformations for specific ligands and discard those that show little or no binding affinity during docking experiments. We developed a set of particular workflow blocks based on AutoDock4.2 system functions and used the 20 ns InhA MD trajectory, described in Material and Methods Section, to perform our experiments. However, our method may be applied to other docking software and different MD trajectories. For this, some blocks of the e-FReDock workflow should be changed to meet the operational needs from the specific virtual screening method. For instance, to execute e-FReDock using AutoDock Vina [8], the AutoGrid process should be eliminated from the workflow, and the AutoDock blocks should be replaced to incorporate the input parameters and the executable program of the AutoDock Vina. Blocks that prepare receptors and ligands, and those which analyze docking results to identify promising conformations remain unchanged.

If a new FFR model is introduced to execute the method proposed, a novel clustering of snapshots should be investigated and generated. The satisfactory results presented in this study was also supported by the high quality of the clustering employed. It means that the set of substrate-binding cavity features is a promising measure of similarity for MD trajectories and it can be extended to other protein/receptors. To generate new clustering of snapshots using substrate-binding cavity features as similarity function, the binding pocket from the new FFR model should be known in advance.

Compared with previous work, the results of this study indicated that the proposed selective method outperforms the original P-SaMI data pattern [48] in the following aspects:

- Accuracy: the P-SaMI validation was obtained from interactions between an FFR model of InhA with only 3,100 snapshots and two ligands, where one of them is NADH, the coenzyme complexed with the InhA enzyme [18, 48]. Conversely, our good accuracy was obtained from interactions between an FFR model with 19,500 snapshots and a set of 74 small molecules based on a cloud-based scientific workflow.
- Self-contained: whereas P-SaMI requires that expert domains provide parameters to identify promising snapshots, our method can select promising docking results and reducing the number of docking experiments, without having any previous information on the protein-ligand interactions. Input parameters required by the user are related to handle the clustering of snapshots and to run AutoDock4.2.

The e-FReDock scientific workflow generated a total of 932,006 Selective Ensemble Docking sub-workflow invocations, among which 238,426 were executed on the Azure cloud platform (around 25 ligands), with the remaining on CIC private cloud. In total 244.66 h were taken from D2 instance, which was the e-SC Server used to run e-FReDock performance tests and e-FReDock experiments, and 1,900.80 h for all 10 D2 v2 instances (e-SC engines). Tables 5 and 6 details the Azure costs regarding the computation and data storage. According to the e-FReDock execution costs ($ 296.11), we can infer that the proposed method also reduced the ensemble docking costs significantly. For instance, if the same experiments were performed for all snapshots of the FFR model, the e-FReDock execution would cost, on average, $ 586.94 for PDB ligands and $ 683.07 for ZINC compounds, taking into account the average reduced percentage from Tables 2 and 3. Likewise, the number of ligands would be decreased by half if the same cost of e-FReDock execution presented in Table 6 had been spent to carry out the ensemble docking experiments on e-FReDock without the method proposed in this study.

**Table 6:** Cost specification spent to run e-FReDock on Azure cloud platform

Cost description	Price (US$)
e-FReDock deployment	10.06
e-FReDock performance tests	22.16
e-FReDock execution	296.11
Blob storage	80.51
File transfer	6.48
Total	415.32

^a^Pricing information from the Azure website as of January 15, 2016 [45]

## Conclusions

This study introduced a method developed to identify groups of snapshots with proper conformational states to accommodate a particular ligand at docking run-time and incorporated it into the e-FReDock cloud-based workflow. A strategic solution was created in the e-SC API to allocate more virtual processors to batches with high priority, and fewer processors to batches with low priority. Experimental results revealed the high accuracy reached by the proposed method for a set of 74 ligands using AutoDock4.2, thereby reducing the model size on average by 53% while keeping the quality of the model by at least 86%. Further experiments were performed for a set of 12 ligands using AutoDock Vina, which also exhibited good accuracy by reducing the percentage of processed snapshots, on average, by 44% and preserving the quality of the RFFR models by at least 83%. The results concluded that, in addition to identifying the best binding affinity of a specific ligand in the FFR model under study, our method was also able to: (1) perform well with different docking methods; (2) select the best docking poses even using a FEB-based selective method; and (3) outperform docking results obtained from the rigid structure of the FFR model.

A natural progression of this study is to work on the overhead imposed when fast docking experiments are executed using more than 8 Azure VMs. Another direction for future research would be to execute our method in larger InhA FFR models and perform docking experiments with more compounds, particularly those already ranked as drug candidates to the FFR model [54] based on ZINCPharmer [52]. It may assist in discovering new potential lead compounds for the InhA enzyme, as well as provide further support to the method proposed in this study.

## Additional file

Additional file 1 **Table S1** - Best FEB values obtained from e-FReDock and cross-docking experiments. (XLSX 11 kb)

## Abbreviations

FEB: Free energy of binding
FFR: Fully-Flexible receptor model
InhA: 2-trans-enoil-ACP (CoA) reductase from Mycobacterium tuberculosis
LGA: Lamarckian genetic algorithm
MD: Molecular dynamics
P-SaMI: Self-adaptive multiple instances
RDD: Rational drug design
RFFR: Reduced Fully-Flexible receptor model
RMSD: Root mean square deviation
TCL: Triclosan
VM: Virtual machine
